# Blockage of SUMO E1 enzyme inhibits ocular lens fibrosis by mediating SMAD4 SUMOylation

**DOI:** 10.1016/j.gendis.2025.101827

**Published:** 2025-08-28

**Authors:** Min Hou, Yujie Ding, Xuan Bao, Liangping Liu, Yulan Wang, Mingxing Wu

**Affiliations:** a State Key Laboratory of Ophthalmology, Guangdong Provincial Key Laboratory of Ophthalmology and Visual Science, Guangdong Provincial Clinical Research Center for Ocular Diseases, Zhongshan Ophthalmic Center, Sun Yat-sen University, Guangzhou, Guangdong 510623, China; b Department of Ophthalmology, Shanghai Eye Diseases Prevention & Treatment Center/Shanghai Eye Hospital, School of Medicine, Tongji University, National Clinical Research Center for Eye Diseases, Shanghai Engineering Research Center of Precise Diagnosis and Treatment of Eye Diseases, Shanghai 200336, China; c Department of Ophthalmology, Gavin Herbert Eye Institute, University of California, Irvine, CA 92617, USA

**Keywords:** Lens capsular fibrosis, Lens epithelial–mesenchymal transition, SMAD4, SUMO E1 inhibitor, SUMOylation

## Abstract

The ocular lens serves as an exemplary biological model for investigating mechanisms of fibrotic disease, particularly through its well-characterized epithelial–mesenchymal transition (EMT) process. In lens capsular fibrosis, lens epithelial cells (LECs) undergo phenotypic transformation mediated by the dysregulation of a complex signaling network. While multiple interconnected pathways have been implicated in this pathogenic process, current therapeutic strategies for anterior subcapsular cataract and postoperative capsular opacification remain predominantly surgical, underscoring the urgent need for targeted pharmacological interventions. SUMOylation, an essential post-translational modification system, orchestrates critical cellular processes, including gene expression, genome integrity, and cell cycle progression. Emerging evidence positions SUMOylation as a critical regulator of EMT in both fibrotic disorders and oncogenesis. Building on these insights, we hypothesized that SUMO-mediated post-transitional modifications may drive LEC transdifferentiation in lens fibrotic pathologies. Our experimental findings demonstrated that elevated global SUMOylation (SUMO1/2/3 conjugates) in human anterior subcapsular cataract specimens correlated with fibrotic progression. Sole SUMO isoform deficiency partially mitigated TGFβ_2_-driven EMT and injury-induced anterior subcapsular cataract. SUMO E1 overexpression enhanced LEC proliferative capacity, migration potential, and EMT progression. Pharmacological SUMO E1 inhibition (ML792) suppressed TGFβ_2_-induced SMAD4 SUMOylation, nuclear translocation, a critical TGFβ/SMAD signaling event. ML792 also eliminated TGFβ_2_-induced LEC EMT and experimental anterior subcapsular cataract. Our results establish SMAD4 SUMOylation as a pivotal molecular switch in lens fibrosis pathogenesis. Employing inhibitory drugs of SUMO conjugation in the years to come has the potential to be a novel therapeutic strategy for fibrotic cataracts.

## Introduction

Fibrotic cataracts always cause premature visual impairment, with anterior subcapsular cataract (ASC) being particularly associated with ocular comorbidities, including uveitis, retinal degeneration, glaucoma, and ocular trauma.^1^, ^2^, ^3^ While cataract surgery remains the primary therapeutic intervention, ASC patients frequently present with anatomical challenges (*e.g.*, shallow anterior chamber and zonular weakness) and chronic ocular inflammation, which collectively increase surgical complexity and elevate postoperative complication risks, such as capsule contraction syndrome, intraocular lens dislocation, and cystoid macular edema.^4^^,^^5^ These clinical challenges underscore the critical need for developing pharmaceutical strategies targeting the molecular mechanisms underlying lens capsular fibrosis.

Epithelial-mesenchymal transition (EMT) contributes a pivotal mechanism driving lens epithelial cell (LEC) transdifferentiation during fibrotic cataractogenesis.^2^ Following ocular trauma or chronic inflammation stimulation, LECs undergo cytoskeletal reorganization, proliferative activation, and up-regulation of mesenchymal markers (particularly, α-smooth muscle actin, [α-SMA]), culminating in fibroblast phenotype acquisition.^2^^,^^6^ Although multiple interconnected signaling pathways (*e.g.*, TGFβ, Wnt, and MAPK) have been implicated in this process,^7^, ^8^, ^9^, ^10^ the definitive regulatory hierarchy governing lens fibrosis remains incompletely characterized.

SUMOylation is a conserved enzymatic post-translational modification involving covalent attachment of small ubiquitin-like modifier (SUMO) proteins to the specific lysine residues on target substrates.^11^, ^12^, ^13^, ^14^ Three types of enzymes involve in the three-step cascade: E1 activation complex (SAE1/UBA2 heterodimer), E2 conjugase (UBC9), and E3 ligase (*e.g.*, RanBP2, PIAS1, and PC2).^11^^,^^15^^,^^16^ The modification exerts dynamically regulatory effects on proteins through multiple mechanisms, including modulation of substrate stability,^17^, ^18^, ^19^ alteration of subcellular localization,^20^^,^^21^ and regulation of DNA-binding affinity.^22^^,^^23^ As a fundamental cellular process, SUMOylation participates in embryogenesis, cell cycle control, and apoptosis,^24^^,^^25^ with growing evidence implicating its dysregulation in oncogenesis,^25^^,^^26^ cardiovascular diseases,^27^^,^^28^ and neurodegenerative disorders.^29^^,^^30^ Notably, SUMOylation modulates EMT through transcription factor regulation (*e.g.*, EZH2 and SnoN SUMOylation suppresses E-cadherin expression in epithelial cancers),^31^^,^^32^ positioning SUMO pathway inhibition as a promising therapeutic candidate in oncology.

In ocular pathophysiology, SUMOylation demonstrates multifaceted regulatory roles: it controls photoreceptor subtype specification via transcription factor modification in photoreceptor development,^22^^,^^33^^,^^34^ maintains p53-dependent heterochromatin stability in retinal pigmental epithelium under oxidative stress in retinal homeostasis,^35^ and regulates lens differentiation (via Pax6 SUMOylation)^22^^,^^23^ and aging process with UBC9 implicated in senile cataractogenesis.^36^ Despite these advances, the functional significance of SUMOylation in lens capsular fibrosis remains underexplored.

Our investigation firstly found elevated global SUMOylation (SUMO1/2/3 conjugates) in human ASC specimens. Sole SUMO paralog deficiency partially mitigated transforming growth factor beta 2 (TGFβ_2_)-driven EMT and experimental ASC. SUMO E1 expression was up-regulated in injury-induced mouse ASC. Overexpression of SUMO E1 promoted LEC proliferation, migration, and EMT. Blockage of SUMOylation via the selective SUMO E1 inhibitor (ML792) effectively attenuated TGFβ_2_-driven LEC EMT and experimental ASC. Moreover, SMAD family member 4 (SMAD4), a critical mediator of TGFβ signaling, underwent SUMOylation mediated by all three SUMO paralogs (SUMO1/2/3). ML792 disrupted TGFβ_2_-induced SMAD4 SUMOylation, nuclear translocation, and accumulation, a key event in TGFβ/SMAD signaling. Together, these results establish SMAD4 SUMOylation as a pivotal regulator of fibrotic cataract pathogenesis. Our findings suggest that SUMOylation inhibition (*e.g.*, via ML792) may represent a novel therapeutic paradigm for fibrotic cataract.

## Materials and methods

### Ethics declaration

This study adhered to the Declaration of Helsinki and institutional guidelines. Human anterior capsulorhexis specimens were collected under protocols approved by the Ethics Committee of Zhongshan Ophthalmic Center, Sun-yet San University (No. R016). All experiments complied with the ARVO Statement for the Use of Animals in Ophthalmic and Vision Research and were approved by the same institution's Animal Use and Care Committee (No. Z2022008 and Z2022036).

### Capsulorhexis specimen collection

The anterior capsulorhexis specimens were collected during standard phacoemulsification from ASC patients and controls (age-related cataract with transparent capsules). Specimens were obtained via continuous curvilinear capsulorhexis, immediately fixed in 100% methanol (15 min), and transferred to phosphate-buffered saline (PBS) for immunofluorescence staining.

### LEC culture and drug treatment

The hTERT-immortalized human LEC line FHL124 (provided by Prof. David Wan-Cheng Li, Zhongshan Ophthalmic Center) was cultured in Dulbecco's Modified Eagle's Medium (#11995500, Gibco Inc., Beijing) supplemented with 10% fetal bovine serum (#10091148, Invitrogen, USA) in a 37 °C incubator with 5% CO_2_. Cells were treated at 30%–40% confluence with drugs. Drug preparation was as follows: TGFβ_2_ (#8406LC, CST, USA) in PBS containing 0.1% bovine serum albumin; ginkgolic acid (#22910, TargetMol, China) and ML792 (#S8697, Selleck, China) in dimethyl sulfoxide (DMSO, #196055, MP Biomedicals, USA).

### *Ex vivo* rat lens culture

Lens from 21 to 28-day-old Sprague–Dawley rats (Sun Yat-Sen University Animal Center) were dissected, free from iris/vitreous, and cultured in serum-free M199 medium (six lenses/dish) containing penicillin (50 IU/mL)/streptomycin (50 mg/mL) (#15140-122, Gibco) overnight. Transparent lenses were treated with DMSO, TGFβ_2_, or ML792 for 7 days. Lenses were fixed for sectioning and staining, and capsules were micro-dissected and stored at −80 °C.

### Injury-induced ASC mouse model and drug administration

Six-to-eight-week-old S129 and C57BL/6J were anesthetized with intraperitoneal 1% pentobarbital sodium (70 μL/10 g) and topical 0.5% proparacaine. Following pupillary dilation (compound tropicamide), a central anterior lens capsule incision was made in the right eye using a 27-gauge needle. Injured eyes received immediate intracameral injection of 1 μL DMSO (PBS) or 10 μM ML792 (PBS) via 30-gauge microsyringe (Hamilton). Mice were allowed to heal for 7 days before sacrifice.

For systematic administration, ML792 (0.75 μg/μL in 5% DMSO/30% polyethylene glycol 300/5% Tween 80) or vehicle (buffer) was intraperitoneally used (7.5 mg/kg) in C57BL/6J mice. Anterior segmental images were captured via slit-lamp microscopy. Eyeballs were harvested for sectioning and staining; lens capsules were analyzed by western blotting and whole-mount staining.

### Transient gene overexpression/knockdown in LECs

For gene overexpression, cDNA from FHL124 LECs was used as the PCR template to amplify coding sequences. Human SUMO genes were cloned into the pCDNA3.1-3xHA vector (#P0160, MiaoLingBio, Wuhan, China) using KPNI (5′) and XbaI (3′) sites, while SUMO/sentrin-specific peptidase 1 (SENP1) gene was cloned into the p3xflag-CMV vector (#P0427, MiaoLingBio) using Hind III (5′) and XbaI (3′) sites. Plasmids were verified by Sanger sequencing and amplified. SUMO- and SENP1-targeting siRNAs were purchased from RiboBio Co., Ltd., Guangzhou, China. Plasmids or siRNAs were transfected into LECs using Lipofectamine 2000™ (#11668019, Invitrogen) for 24 h before experiments or cell harvest. All cloning oligos listed in Tables S1 and S2 were obtained from Tsingke Technologies (Guangzhou, China).

### Establishment of stable cell lines via lentivirus infection

Human SAE1, UBA2, and wild-type (WT)/mutant SMAD4 cDNAs were cloned into the pLVX-IRES-Puro-3xFlag vector (#VT8006, Youbio, China) using EcoRI (5′) and XboI (3′) sites. For shRNA knockdown, oligonucleotide pairs were annealed and ligated into the pLKO.1-TRC plasmid at AgeI (5′) and EcoRI (3′) sites.

Lentivirus was produced by co-transfecting HEK-293FT cells with the target plasmid, psPAX2 (packaging), and pMD2.G (envelope) plasmid. A stable cell line was selected using 1.25 μg/mL puromycin and validated by quantitative reverse transcription PCR and western blotting. Oligo sequences are all listed in Tables S1 and S2.

### Histology and immunohistochemistry analysis

Eye balls were fixed in FAS eye fixation solution (#G1109, Servicebio, Wuhan, China) and rat lenses in 4% paraformaldehyde for 24 h. Tissues were dehydrated through an ethanol series, cleared in xylene, and paraffin-embedded. Serial 4-μm sections through the optic disk (at least three sections/eye) were prepared for hematoxylin-eosin staining. For immunohistochemistry, antigen retrieval was performed in 10 mM sodium citrate buffer (95 °C, 30 min). Sections were treated with 3% H_2_O_2_ for 10 min, blocked with normal goat serum (room temperature, 1 h), and then incubated with primary antibodies (1:100) at 4 °C overnight. After PBS washing, sections were incubated with horseradish peroxidase-conjugated secondary antibodies (room temperature, 50 min), developed with 3,3′-diaminobenzidin, and imaged using a Tissue FAX confocal microscope (Q+, TissueGnostics, Austria).

### Cell proliferation analysis

Cell proliferation was assessed using the CCK-8 kit (# CK04, Dojindo Laboratories, Kumamoto, Japan). FHL124 LECs (3 × 10^3^ cells/well) were seeded in 96-well plates for 8 h, serum-starved in fetal bovine serum-free Dulbecco's Modified Eagle's Medium, and then treated with 0.1% DMSO or 10 μM ML792 for 24 or 48 h. After treatment, 10 μL CCK-8 solution was added per well and incubated at 37 °C for 1 h. Absorbance at 450 nm was measured using a microplate reader (Elx800; Bio-Tek Inc., North Brunswick, USA). All conditions were tested in triplicate across three independent experiments.

Proliferation of SAE1/UBA2-overexpressed LECs was assessed using the BeyoClick™ EdU kit (#C0078S; Beyotime, Shanghai, China). Cells were seeded in 12-well plates, cultured overnight, and then pulsed with 10 μM EdU for 2 h. After fixation and permeabilization, cells were sequentially stained with the click reaction mixture and Hoechst. Fluorescence images were acquired using a microscope.

### Co-immunoprecipitation assay

Cells were lysed in 0.5% NP-40 buffer (10 mM Tris-Cl, pH 7.4, 150 mM NaCl, 0.5% NP-40, 10% glycerol) containing protease inhibitors (#P2714, Sigma–Aldrich, Missouri, USA) on ice for 5 min. Lysate (2 mg) was precleared with control IgG (#2729, #53484, CST) at 4 °C for 2 h. Immunoprecipitation was performed at 4 °C overnight using anti-Flag antibody/Nano-Agarose beads (#FNM-25-500, NuoyiBio, Tianjin, China), anti-SUMO1 or anti-HA antibody with protein A/G Magnetic beads (#HY-K0202, MedChemExpress, New Jersey, USA). Beads were washed three times with immunoprecipitation buffer, resuspended in SDS sample buffer, and analyzed by SDS-PAGE. Western blotting was performed as described.

### Others

Immunofluorescence, PCR, western blotting, wound-healing, and Transwell migration assays were performed as previously described.^36^, ^37^, ^38^ For immunofluorescence staining, cells were labeled with iFluor™ 488 phalloidin (#40735ES75, YEASEN, China) for F-actin and DAPI (50 ng/mL, #10236276001, Roche, Basel, Switzerland) for nuclei. Primers and antibodies are listed in Table S3 and Table S4, respectively.

### Statistical analysis

Data were analyzed with SPSS 17.0 (SPSS Inc., Chicago, Illinois, USA). Results shown represent at least three independent experiments. Normality was assessed by the Kolmogorov–Smirnov test. Significance (*P* < 0.05) was determined by two-tailed Student's *t*-test (two groups) or one-way analysis of variance (ANOVA) with Bonferroni post-hoc test (multiple groups). Significance levels: ∗*P* < 0.05, ∗∗*P* < 0.01, and ∗∗∗*P* < 0.001.

## Results

### SUMOylation drives EMT in human ASC

**Figure 1:**
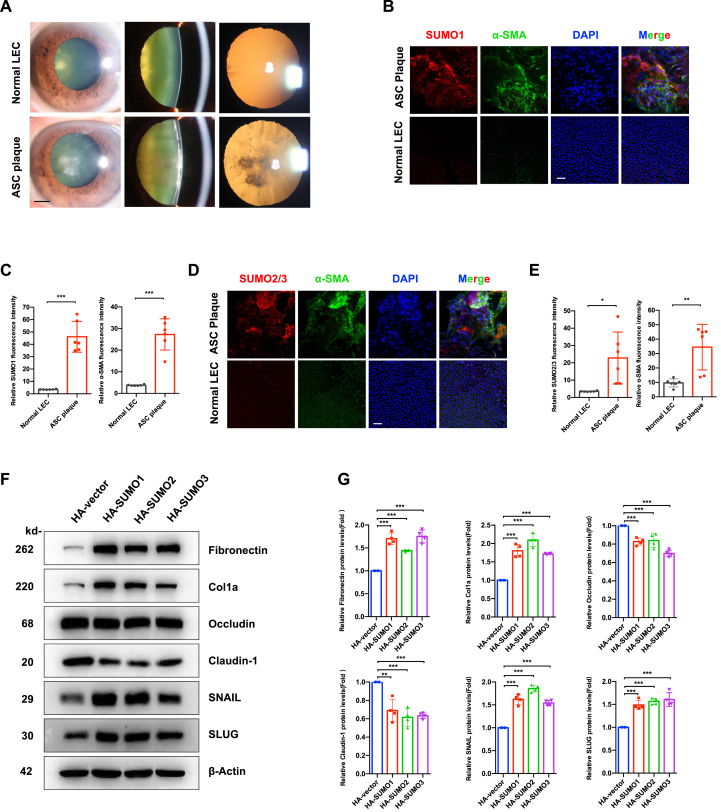
SUMOylation is elevated in human anterior subcapsular cataract (ASC) and drives the epithelial–mesenchymal transition (EMT) of lens epithelial cells (LECs). **(A)** Representative anterior segment photographs of transparent capsules and ASC plaques in human patients. Scale bar: 2 mm. **(B)** Immunofluorescence staining of lens epithelium from ASC patients and controls. SUMO1 (red), α-SMA (green), and DAPI (nuclear counterstain, blue). Scale bar: 50 μm. **(C)** Quantification analysis of SUMO1 and α-SMA fluorescence intensity from (B). Two random regions per capsule were analyzed (*n* = 3 capsules/group). Statistical significance was determined by an unpaired Student's *t*-test. ∗∗∗*P* < 0.001. **(D)** Immunofluorescence co-staining of SUMO2/3 (red), α-SMA (green), and DAPI (nuclear, blue) in lens epithelium. Scale bar: 50 μm. **(E)** Quantification of SUMO2/3 and α-SMA fluorescence intensity from (D). Data were analyzed as in (C). ∗*P* < 0.05 and ∗∗*P* < 0.01. **(F)** Western blotting analysis of EMT markers in FHL124 LECs transfected with HA-tagged vector and SUMO isoforms. Cells were harvested 24 h post-transfection. **(G)** Densitometric quantification of (F). Data were normalized to β-actin loading control. Statistical analysis was performed by one-way ANOVA with Bonferroni post-hoc test. ∗∗*P* < 0.01 and ∗∗∗*P* < 0.001.

Slit-lamp examination revealed characteristic lens subcapsular opacity in ASC specimens, contrasting with transparent lens capsules from control donors (Fig. 1A). Immunofluorescence analysis demonstrated pathological restructuring of LECs in ASC specimens, manifested by the hallmark features: i) multilayered cell aggregates with spindle-shaped nuclei replacing the normal monolayer of cuboidal epithelium adhere to lens capsule; ii) significant up-regulation of α-SMA expression (*P* < 0.001 and *P* < 0.01 versus controls) (Fig. 1C, E); iii) enhanced global protein conjugation by SUMO1 (*P* < 0.001) and SUMO2/3 (*P* < 0.05) (Fig. 1B–E).

Mechanistic investigation using gain-of-function models showed that transient overexpression of individual SUMO paralogs (SUMO1/2/3) in human LECs (Fig. S1) induced reduction in tight junction proteins (occludin and claudin-1), increase in fibronectin and collagen type I (Col1a), up-regulation of EMT transcription factors SNAIL and SLUG (*P* < 0.01 and *P* < 0.001 versus vector controls) (Fig. 1F and G).

### SUMO isoform deficiency partially mitigates TGFβ_2_-driven EMT and experimental ASC

**Figure 2:**
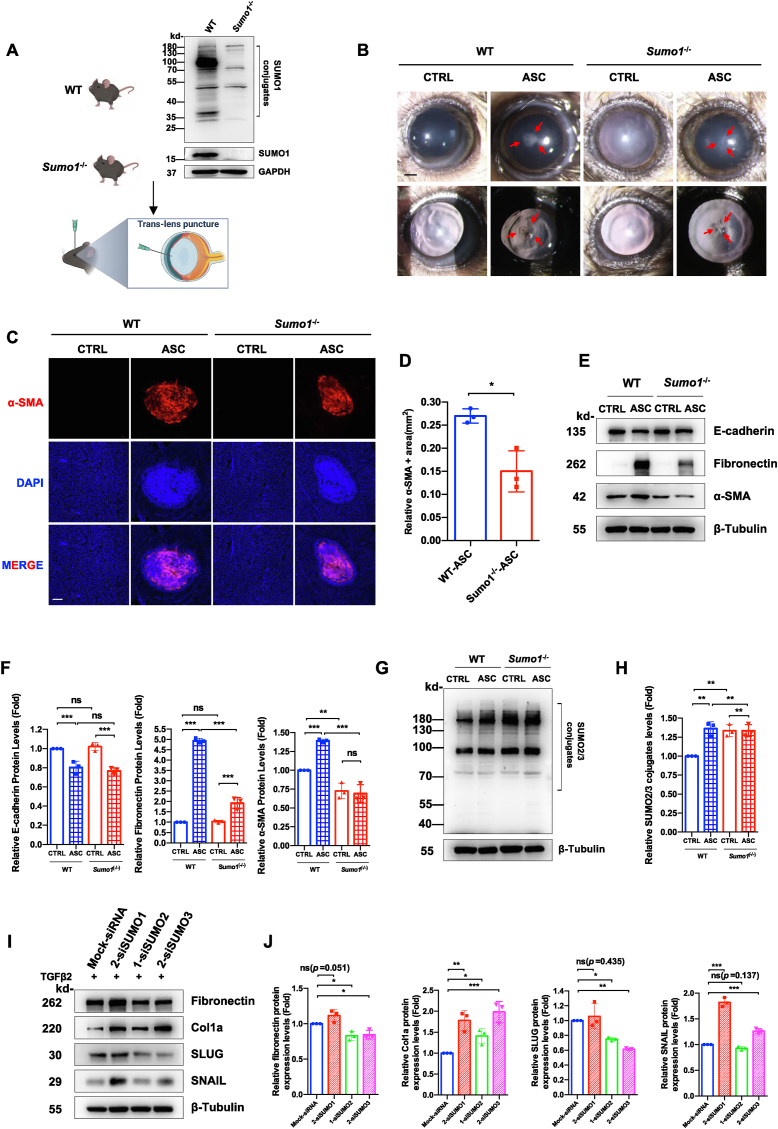
SUMO isoform-specific deficiency partially attenuates TGFβ_2_-induced epithelial–mesenchymal transition (EMT) and anterior subcapsular cataract (ASC) progression. **(A)** Immunoblot of free SUMO1-conjugated proteins in lens capsules from wild-type (WT) and *Sumo1*^*−/−*^ (S129 background) mice. Schematic illustration of the murine ASC induction protocol: Anterior capsule puncture with a 27-gauge needle followed by a 7-day healing phase. Injured eyes (ASC) versus contralateral controls (CTRL). *n* = 6–8 per group. **(B)** Representative slit-lamp images of anterior subcapsular plaques (red arrow) in injured eyes (ASC) versus uninjured eyes (CTRL) in WT and *Sumo1*^*−/−*^ (S129 background) mice at day 7. Scale bar: 0.5 mm. **(C)** α-SMA (red) and DAPI (nuclear, blue) in whole-mount immunofluorescence of anterior capsules from (B). Scale bar: 100 μm. **(D)** Quantification of α-SMA^+^ areas from (C). *n* = 3 biological replicates per group. Unpaired Student's *t*-test; ∗*P* < 0.05. **(E**–**H)** Western blotting analysis of epithelial and EMT markers (E-cadherin, fibronectin, and α-SMA) and SUMO2/3 conjugates in lens epithelium, followed by densitometric quantification normalized to β-tubulin. One-way ANOVA followed by Bonferroni's correction; ns, not significant; ∗∗*P* < 0.01 and ∗∗∗*P* < 0.001. **(I)** siRNA-mediated SUMO isoform knockdown in FHL124 lens epithelial cells (LECs). Cells were transfected with non-targeting siRNA (Mock) or SUMO1/2/3-specific siRNAs for 24 h, followed by TGFβ_2_ (10 ng/mL) treatment for 24 h. Western blotting analysis of EMT markers fibronectin, collagen I, SLUG, and SNAIL proteins in the LECs was performed. β-Tubulin served as the loading control. **(J)** Quantification of (I) normalized to β-tubulin. One-way ANOVA followed by Bonferroni post-hoc test; ns, not significant; ∗*P* < 0.05, ∗∗*P* < 0.01, and ∗∗∗*P* < 0.001.

Genetic ablation of *Sumo1* in S129 mice resulted in complete loss of SUMO1 protein expression in LECs without compromising ocular development, as evidenced by comparable lens/retina morphology and E-cadherin levels between *Sumo1*^*−/−*^ and WT mice (Fig. 2A; Fig. S2B–S2D).

In our needle-induced ASC models (Fig. 2A and B), *Sumo1*^*−/−*^ mice exhibited a reduction in fibrotic plaque area by slit-lamp imaging, a decrease in α-SMA positive lesion size via immunofluorescence (*P* = 0.012 *vs*. WT-ASC) (Fig. 2B–D), and down-regulation of fibronectin (*P* < 0.001 *vs*. WT-ASC), and reduction in α-SMA (*P* < 0.001 *vs*. WT-ASC) by immunoblotting (Fig. 2E and F). Notably, *Sumo1* deficiency resulted in compensatory SUMO2/3 hyperactivation, with increased SUMO2/3 conjugation in naive LECs (*P* < 0.01 *vs*. WT) and elevated SUMO2/3 levels post-injury (*P* < 0.01 *vs*. WT-ASC) (Fig. 2G and H).

Given the lethality of SUMO1/2 knockout cells in our previous experiment, we employed siRNA-mediated isoform-specific knockdown (Fig. S3A). Paradoxically, *SUMO1* silencing failed to suppress TGFβ_2_-induced fibronectin (*P* = 0.051 *vs*. mock-siRNA) and SLUG (*P* = 0.435), while resulting in increased collagen I (*P* < 0.01) and SNAIL (*P* < 0.001) expression (Fig. 2I and J). *SUMO1* silencing also induced increased SUMO2/3 conjugation (Fig. S3D and S3E). Similarly, SUMO2 and SUMO3 knockdown dysregulated EMT markers, resulting in increased collagen I (*P* < 0.05 and *P* < 0.001 *vs*. mock-siRNA, respectively) and SNAIL (*SUMO3 vs*. mock-knockdown, *P* < 0.001) and decreased fibronectin (both *P* < 0.05) and SLUG expression (*P* < 0.05 and *P* < 0.01, respectively) (Fig. 2I and J). Simultaneously, SUMO2/3 deficiency caused increased SUMO1 conjugates (both *P* < 0.05) (Fig. S3B and S3C).

### SUMO E1 exhibits dynamic changes in the mouse ASC model and promotes fibrotic transformation of LECs

**Figure 3:**
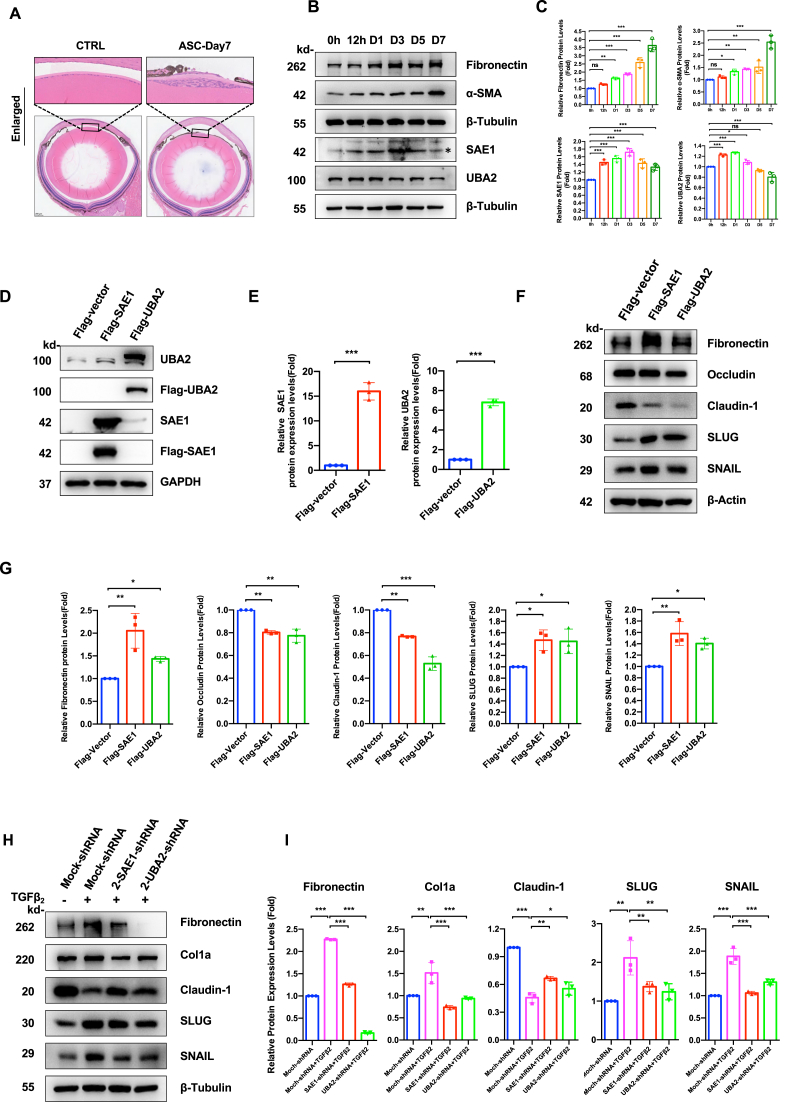
SUMO E1 exhibits dynamic changes in the mouse anterior subcapsular cataract (ASC) model and promotes fibrotic transformation of lens epithelial cells (LECs). **(A)** Histopathological analysis of wild-type (WT) C57BL/6J mouse lenses 7 days post-capsular injury. Hematoxylin-eosin staining revealed capsular fibrosis in injured eyes (ASC) versus contralateral controls (CTRL). Scar bar: 200 μm. **(B)** Time-course immunoblot analysis of epithelial–mesenchymal transition (EMT) markers (fibronectin and α-SMA) and SUMO E1 components (SAE1/UBA2) in lens epithelium following injury (0 h–7 d post-operation) performed on WT C57BL/6J mice as indicated in (A). β-Tubulin served as the loading control. **(C)** Densitometric quantification of (B). *n* = 6–8 biological replicates per time point. One-way ANOVA with Bonferroni correction; ns, not significant; ∗*P* < 0.05, ∗∗*P* < 0.01, and ∗∗∗*P* < 0.001. **(D)** FHL124 LECs were established to overexpress empty vector control, Flag-tagged SAE1, and Flag-tagged UBA2 via the lentivirus infection plasmid system. Immunoblot analysis confirmed exogenous SAE1 and UBA2 protein expression. GAPDH served as the loading control. **(E)** Quantification of overexpression efficiency from (D). Unpaired Student's *t*-test; ∗∗∗*P* < 0.001 versus empty vector control. **(F)** Analysis of epithelial and EMT markers in engineered LECs from (D). β-Actin served as the loading control. **(G)** Quantification of (F). One-way ANOVA with Bonferroni post-hoc test; ∗*P* < 0.05, ∗*P* < 0.01, and ∗∗∗*P* < 0.001. **(H)** ShRNA-mediated SAE1/UBA2 knockdown in LECs with or without treatment of TGFβ_2_ (10 ng/mL, 24 h). Western blotting analysis of EMT markers fibronectin, collagen I, SLUG, and SNAIL, and epithelial marker claudin-1 in the LECs. β-Tubulin served as the loading control shown. **(I)** Quantification analysis of (H). One-way ANOVA with Bonferroni correction; ns, not significant; ∗*P* < 0.05, ∗∗*P* < 0.01, and ∗∗∗*P* < 0.001.

Time-course analysis of SUMO E1 components (SAE1/UBA2) in our murine ASC model (Fig. 3A) revealed biphasic expression kinetics. SAE1 and UBA2 protein levels peaked at day 3 post-injury (both *P* < 0.001 *vs*. baseline), and subsequently, UBA2 protein level declined, and SAE1 protein still displayed a higher level (*P* < 0.001) (Fig. 3B and C).

Functional validation through genetic manipulation demonstrated SUMO E1's pivotal role in EMT regulation. Gain-of-function studies (Fig. S4A and S4B; Fig. 3D and E) demonstrated that overexpression of SAE1/UBA2 caused significant increase in fibronectin (*P* < 0.01 and *P* < 0.05), SNAIL (*P* < 0.01 and *P* < 0.05), and SLUG (both *P* < 0.05) expression, and induced a reduction of occludin (both *P* < 0.01) and claudin-1 (*P* < 0.01 and *P* < 0.001) protein levels (Fig. 3F and G).

In loss-of-function studies (Fig. S4C–S4F), either SAE1 or UBA2 knockdown displayed decrease in both global SUMO1 and SUMO2/3 conjugations (Fig. S5A–S5D). Simultaneously, E1 knockdown caused suppression of TGFβ_2_-induced fibronectin (both *P* < 0.001 *vs*. mock-shRNA), collagen type I (both *P* < 0.001), SLUG (both *P* < 0.01), and SNAIL (both *P* < 0.001), and reversed TGFβ_2_-induced claudin-1 reduction (*P* < 0.01 and *P* < 0.05, respectively) (Fig. 3H and I).

### SUMO E1 enzyme drives proliferative capacity and invasion in LECs

**Figure 4:**
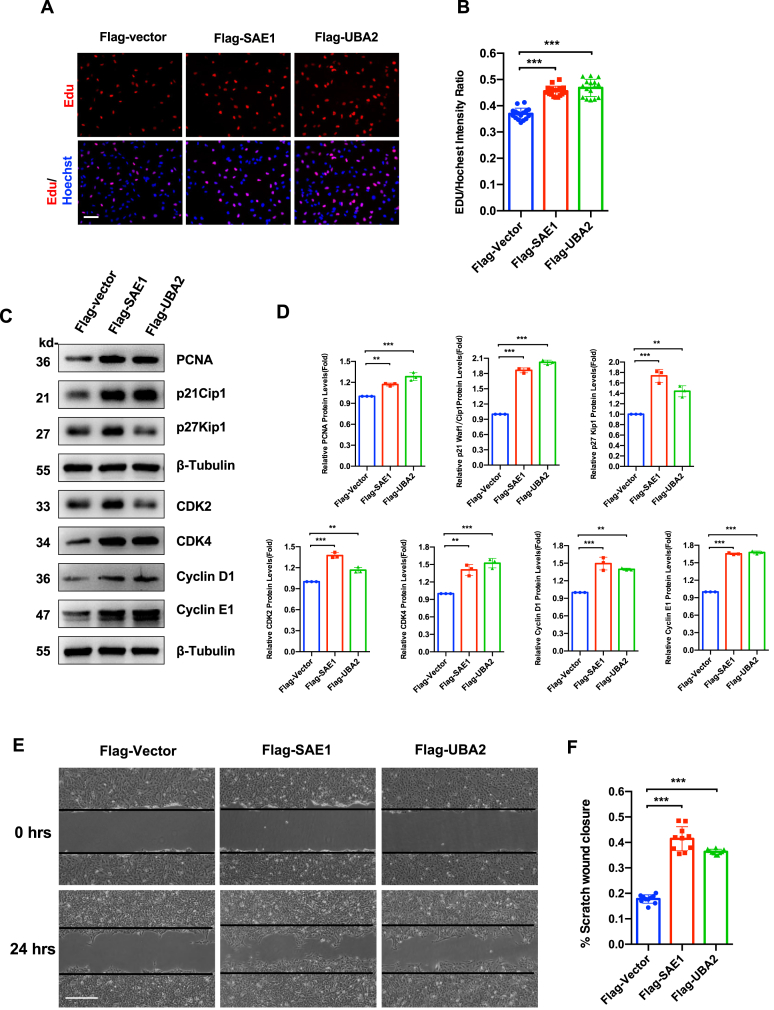
SUMO E1 promotes proliferative and invasive phenotypes in human lens epithelial cells (LECs). **(A)** Edu/Hoechst proliferation assay in FHL12.4 LECs stably expressing empty vector, Flag-tagged SAE1, or Flag-tagged UBA2 (expression was detailed in Fig. 3D). Nuclei were counterstained with Hoechst (blue), and proliferating cells were labeled by EdU (red). Scar bar: 100 μm. **(B)** Quantification of Edu^+^ proliferative cells from (A). Data were normalized to the total nuclei count. One-way ANOVA with Bonferroni post-hoc test; ∗∗∗*P* < 0.001. **(C)** Immunoblot analysis of cell cycle regulators in engineered LECs: proliferation marker PCNA, cyclin-dependent kinase inhibitors p21Cip1 and p27Kip1, and cell cycle engines, CDK2, CDK4, cyclin D1, and cyclin E. β-Tubulin served as the loading control. **(D)** Densitometric quantification of (C). Data were expressed as fold-change versus vector control. One-way ANOVA with Bonferroni correction; ∗∗*P* < 0.01 and ∗∗∗*P* < 0.001. **(E)** Scratch wound healing assay. Confluent monolayers of engineered LECs were wounded with 20 μL pipette tips (0 h), with migration progression monitored at 24 h. Representative phase-contrast images show wound closure dynamics. Scale bar: 500 μm. **(F)** Quantification analysis of the wound closure rates from (E) (calculated as percentage of wound area reduction at 24h vs. baseline. One-way ANOVA with Bonferroni post-hoc test; ∗∗∗*P* < 0.001.

Functional characterization of SUMO E1-overexpressing LECs (established in Fig. 3D) revealed profound alterations in proliferative and migratory behavior. Edu staining was significantly enhanced in engineered LECs (both *P* < 0.001 *vs*. empty vector controls) (Fig. 4A and B). Cell cycle regulator profiling by immunoblot analysis showed up-regulation of proliferation marker proliferating cell nuclear antigen (PCNA), cyclin-dependent kinase inhibitors p21Cip1 and p27Kip1, and cell cycle engines, cyclin-dependent kinase 2 (CDK2), CDK4, cyclin D1, and cyclin E (all *P* < 0.01 or 0.001 *vs*. empty vector controls) (Fig. 4C and D). Scratch wound healing assay demonstrated about 2-fold enhanced planar migration capacity (both *P* < 0.001 *vs*. empty vector controls). Transwell invasion assay showed comparable transmigration capacity across groups (Fig. S6).

### Pharmacological inhibition of SUMO E1 attenuates TGFβ_2_-driven EMT and prevents ASC progression

**Figure 5:**
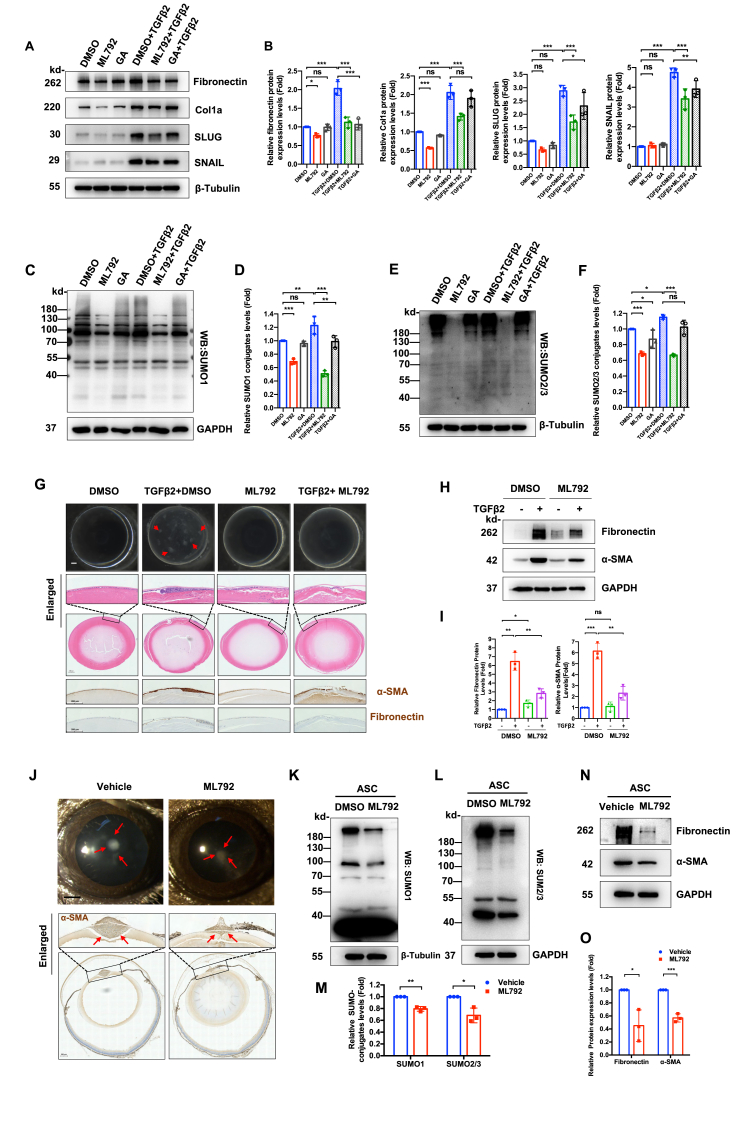
Pharmacological inhibition of SUMO E1 attenuates TGFβ_2_-driven epithelial–mesenchymal transition (EMT) and prevents anterior subcapsular cataract (ASC) progression. **(A)** FHL124 lens epithelial cells (LECs) were treated with 0.1% DMSO, 10 μM ML792, and 10 μM Ginkgolic acid (GA), along with or without the treatment of 10 ng/mL TGFβ_2_ for 24 h. Immunoblot analysis of EMT markers, fibronectin, Collagen I, SLUG, and SNAIL proteins was performed. β-Tubulin served as the loading control. **(B)** Densitometric quantification of (A). One-way ANOVA with Bonferroni correction; ns, not significant; ∗*P* < 0.05, ∗∗*P* < 0.01, and ∗∗∗*P* < 0.001. **(C–F)** Global SUMOylation profiling in treatment groups from (A). (C) SUMO1 conjugate immunoblot. (E) SUMO2/3 conjugate immunoblot. (D, F) Quantification analysis of SUMOylation levels (normalized to GAPDH and β-tubulin). One-way ANOVA with Bonferroni correction; ns, not significant; ∗*P* < 0.05, ∗∗*P* < 0.01, and ∗∗∗*P* < 0.001. **(G)***Ex vivo* rat lens organ culture model. Macroscopic lens opacity assessment after 7-day treatments: vehicle (0.1% DMSO), TGFβ_2_ (10 ng/mL), ML792 (10 μM), and TGFβ_2_ plus ML792. Bottom: histopathological analysis (hematoxylin-eosin staining) and fibrotic marker immunohistochemistry staining (fibronectin/α-SMA). Scar bar: 200 μm. **(H, I)** Immunoblot validation of fibrotic markers in lens epithelium from (G). GAPDH served as the loading control. One-way ANOVA with Bonferroni post-hoc test; ns, not significant; ∗*P* < 0.05, ∗∗*P* < 0.01, and ∗∗∗*P* < 0.001. **(J)***In vivo* therapeutic efficacy in C57BL/6J mice: intracameral injection into the anterior ocular chamber with vehicle (0.1% DMSO diluted in PBS) and ML792 (10 μM diluted in PBS) administered immediately post-capsular injury (*n* = 6 biological replicates/group). 7-day endpoints: slit-lamp imaging (red arrow indicated plaques; scale bar: 0.5 mm) and immunohistochemistry staining of α-SMA protein (scale bar: 200 μm). **(K, L)** Immunoblot analysis of SUMOylation status in murine lens epithelium from the therapeutic intervention groups described in (J). **(M)** Densitometric quantification of SUMO conjugation levels (SUMO1, SUMO2/3). Data were normalized to β-tubulin and GAPDH. Unpaired Student's *t*-test; ∗*P* < 0.05 and ∗∗∗*P* < 0.01. **(N, O)** Immunoblot analysis of fibrotic markers (fibronectin/α-SMA) in murine lens epithelium from (J), followed by densitometric quantification. GAPDH served as the loading control. Unpaired Student's *t*-test; ∗*P* < 0.05 and ∗∗∗*P* < 0.001.

Pharmacological targeting of the SAE1/UBA2 complex with a selective inhibitor ML792 (SUMO E1-specific) and pan-SUMOylation inhibitor ginkgolic acid revealed distinct therapeutic profiles. ML792 demonstrated broad-spectrum inhibition of TGFβ_2_-induced fibronectin, collagen I, SLUG, and SNAIL expression (all *P* < 0.001) (Fig. 5A and B). Concurrently, it effectively suppressed both SUMO1 and SUMO2/3 conjugation regardless of TGFβ stimulation status (all *P* < 0.001) (Fig. 5C–F). While ginkgolic acid exhibited isoform-selective effects on SUMOylation inhibition, it had a suppression of SUMO1 conjugation (*P* < 0.01), relatively weaker than ML792, while maintaining SUMO2/3 conjugation (*P* = 0.433) under TGFβ_2_ stimulation (Fig. 5C–F). It partially suppressed TGFβ_2_-induced EMT by inhibiting fibronectin (*P* < 0.001), SLUG (*P* < 0.05), and SNAIL (*P* < 0.01) expression (Fig. 5A and B).

Both compounds maintained post-translational specificity, showing no off-target effects on acetylation/ubiquitination (Fig. S7A–S7D). To furtherly validate the potential off-target effects on other pathways, we overexpressed SENP1 (a SUMO-specific protease 1) in LECs as a genetic approach to mimic SUMO E1 inhibitory effects. SENP1 overexpression caused a detectable reduction in EMT marker expression and in the global SUMO1- and SUMO2/3-mediated conjugation (Fig. S8A–S8F). SENP1 knockdown did not obviously affect TGFβ_2_-induced EMT, suggesting compensatory deSUMOylation of other SENPs (Fig. S9).

Cytotoxicity assessment revealed that ML792 (10 μM) exhibited no acute toxicity in human LECs during 24 h treatment (CCK-8 assay in Fig. S7E). ML792's superior efficacy versus ginkgolic acid justified its selection for subsequent studies. Next, we evaluated ML792's therapeutic efficacy in *ex vivo* models. In TGFβ_2_-stimulated rat lens cultures, ML792 administration significantly reduced global SUMOylation levels (Fig. S10) and potentially inhibited anterior subcapsular plaque (Fig. 5G). Histopathological analysis revealed that ML792 prevented TGFβ_2_-induced multilayer cellular reorganization at the anterior capsule, as evidenced by hematoxylin-eosin staining (Fig. 5G). Immunohistochemistry and immunoblot analyses further confirmed ML792's ability to suppress TGFβ_2_-driven up-regulation of fibronectin (*P* < 0.01) and α-SMA (*P* < 0.01) proteins in lens capsules (Fig. 5G–I).

To validate *in vivo* relevance, ML792 was administered via two routes in murine models. Systemic delivery (intraperitoneally) caused no structural abnormalities in ocular or major organs (Fig. S11). Local intracameral administration of ML792 post-injury exhibited retinal architecture preservation, confirmed by histological analysis (Fig. 5J). It effectively reduced global SUMO1- and SUMO2/3-mediated conjugation (*P* < 0.01 and *P* < 0.05 *vs*. vehicle groups, respectively) (Fig. 5K–M). Fibronectin and α-SMA proteins in LECs adhered to capsules were also significantly attenuated (*P* < 0.05 and *P* < 0.001, respectively) (Fig. 5N and O).

These results collectively demonstrate that ML792, a selective SUMO E1 inhibitor, effectively inhibits TGFβ_2_/injury-induced EMT in LECs through precise blockade of global SUMOylation, while maintaining an excellent safety profile across administration routes.

### ML792 disrupts SMAD4 SUMOylation-dependent nuclear trafficking in TGFβ signaling

To delineate the interplay between SUMOylation and canonical TGFβ signaling in lens fibrosis, we employed a dual pharmacological/genetic approach in human LECs.

**Figure 6:**
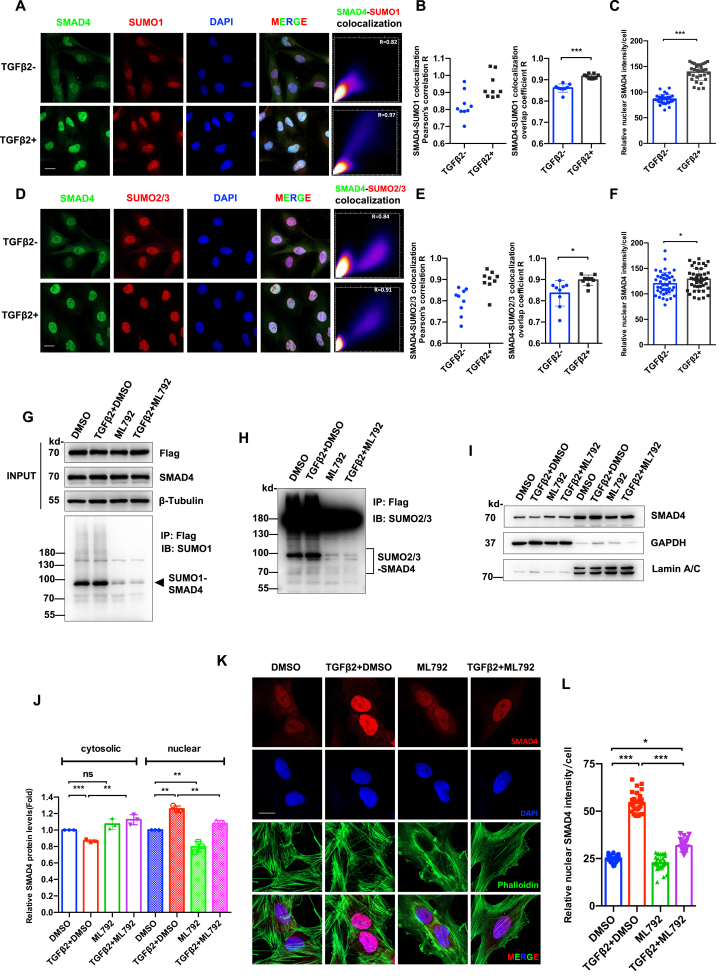
ML792 disrupts SMAD4 SUMOylation-dependent nuclear translocation in TGFβ_2_-stimulated lens epithelial cells (LECs). **(A**–**F)** FHL124 LECs were treated with or without TGFβ2 (10 ng/mL, 2 h). Triple immunofluorescence staining of SMAD4 (green), SUMO1 (red)/SUMO2/3 (red), and DAPI (nuclei, blue) shows spatiotemporal dynamics of SMAD4-SUMO colocalization. (A, D) SMAD4-SUMO1/SUMO2/3 immunofluorescence staining and colocalization scatterplot. (B, E) Pearson's *r* analysis of colocalization performed by Image J. *n* = 9 replicates per group. (C, F) Quantification of nuclear SMAD4 intensity. *n* = 30 cells in (C) and *n* = 44 cells in (F). Unpaired Student's *t*-test; ∗*P* < 0.05 and ∗∗∗*P* < 0.001. **(G, H)** Flag-SMAD4 immunoprecipitation in engineered FHL124 LECs overexpressing Flag-SMAD4. Treatments were 0.1% DMSO, TGFβ_2_ (10 ng/mL), ML792 (10 μM), or their combination for 2 h. (G, H) Whole-cell lysates were blotted with anti-Flag and anti-SMAD4 (INPUT). Cell lysates were immunoprecipitated with anti-Flag, followed by SUMO1 immunoblotting (G) and SUMO2/3 immunoblotting (H). **(I, J)** Subcellular fractionation analysis. (I) Immunoblots of cytoplasmic/nuclear SMAD4 after 8 h treatments in FHL12.4 LECs. (J) Quantification was normalized to GAPDH (cytoplasm) and lamin A/C (nucleus). One-way ANOVA with Bonferroni correction; ns, not significant; ∗∗*P* < 0.01 and ∗∗∗*P* < 0.001. **(K, L)** SMAD4 nuclear translocation analysis. (K) Triple immunofluorescence staining SMAD4 (red), F-actin (Phalloidin, green), and DAPI (nuclei, blue) in LECs treated as indicated in (I). Scar bar: 20 μm. (L) Nuclear SMAD4 fluorescence intensity quantification. *n* = 30 cells per group. One-way ANOVA with Bonferroni post-hoc test; ∗*P* < 0.05 and ∗∗∗*P* < 0.001.

ML792 preserved total SMAD2/3/4 protein levels and maintained TGFβ_2_-induced SMAD2/3 phosphorylation (Fig. S12A and S12B). This suggests that SUMOylation acts downstream of canonical SMAD phosphorylation. Immunofluorescence co-staining of SMAD4 and SUMO isoforms with quantitative colocalization analysis (Pearson's coefficient: 0.82 ± 0.08 for SMAD4-SUMO1 and 0.79 ± 0.06 for SMAD4-SUMO2/3) in naive LECs (Fig. 6A, B, D, E) revealed spatiotemporal interaction hotspots between SMAD4 and SUMO paralogs. The overlap significantly increased after TGFβ_2_ treatment (Pearson's overlap coefficient: 0.86 ± 0.19 *vs*. 0.91 ± 0.01 for SMAD4-SUMO1, *P* < 0.001; 0.84 ± 0.06 *vs*. 0.90 ± 0.02 for SMAD4-SUMO2/3, *P* < 0.001) (Fig. 6B, E), with nuclear SMAD4 foci showing higher colocalization density after TGFβ_2_ treatment (*P* < 0.001, *P* < 0.05, respectively) (Fig. 6C and F).

Co-immunoprecipitation assays in SMAD4-and SUMO-overexpressing LECs demonstrated that SMAD4 was a multi-SUMOylation substrate, and all three SUMO isoforms could conjugate to SMAD4 in human LECs (Fig. S13).

ML792 demonstrated potent suppression of TGFβ_2_-induced SMAD4 SUMOylation mediated by SUMO1 and SUMO2/3 (Fig. 6G and H). Mechanistically, immunoblot analysis revealed that ML792 disrupted SMAD4 nucleocytoplasmic shuttling, increasing cytoplasmic SMAD4 retention (*P* < 0.01) and decreasing nuclear SMAD4 accumulation (*P* < 0.01) (Fig. 6I and J). Quantitative immunofluorescence analyses validated that ML792 hindered TGFβ_2_-induced SMAD4 nuclear shuttling (*P* < 0.001, TGFβ_2_ + DMSO *vs*. TGFβ_2_ + ML792) (Fig. 6K and L).

These data established SUMOylation as a critical post-phosphorylation regulatory node in TGFβ signaling, selectively modifying SMAD4 to mediate its nuclear trafficking and transcriptional competence, rather than affecting upstream SMAD activation events.

### SUMOylation site mutagenesis disrupts SMAD4-mediated fibrotic signaling

**Figure 7:**
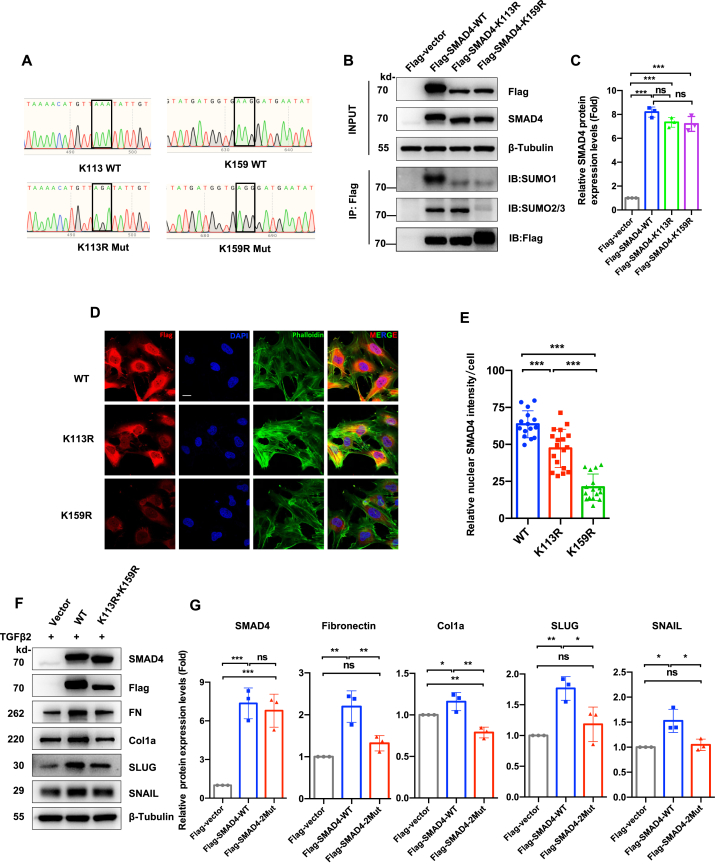
SUMOylation site mutagenesis abolishes SMAD4-mediated epithelial–mesenchymal transition (EMT) in TGFβ_2_-stimulated lens epithelial cells (LECs). **(A)** Sanger sequencing validation of SMAD4 mutants. WT, wild-type; K113R, Lys113→Arg; K159R, Lys159→Arg. The black frames indicate WT and mutated codons. **(B, C)** SUMOylation capacity analysis in SMAD4 mutants. (B) FHL124 LECs stably overexpressed empty vector and flag-SMAD4 variants treated with TGFβ_2_ (10 ng/mL, 2 h). Whole-cell lysates were immunoblotted with anti-Flag and anti-SMAD4. β-Tubulin served as the loading control. The cell lysates were immunoprecipitated with anti-Flag nano beads, followed by immunoblotting for SUMO1, SUMO2/3, and Flag antibody. (C) Quantification of SMAD4 expression (Input lysates). One-way ANOVA with Bonferroni correction; ns, not significant; ∗∗∗*P* < 0.001. **(D, E)** SMAD4 nuclear translocation analysis. (D) Triple fluorescence imaging of Flag (SMAD4, red), F-actin (phalloidin, green), and DAPI (nuclei, blue) in engineered LECs treated with TGFβ_2_ (10 ng/mL, 2 h). (E) Nuclear SMAD4 intensity quantification (*n* = 15–18 cells/group). One-way ANOVA with Bonferroni post-hoc test; ∗∗∗*P* < 0.001. **(F, G)** Functional consequence of double site mutant (K113 plus 159R) SMAD4 protein. (F) EMT marker immunoblotting 24 h after TGFβ_2_ treatment in human LECs overexpressing empty vector, WT Flag-tagged SMAD4, or double site mutant Flag-tagged SMAD4. (G) Densitometric analysis from (F). β-Tubulin served as the loading control. One-way ANOVA followed by Bonferroni correction; ns, not significant; ∗*P* < 0.05. ∗∗*P* < 0.01, and ∗∗∗*P* < 0.001.

To verify this SUMOylation-dependent regulatory axis for intercepting fibrotic signaling, we furtherly reconstructed SUMOylation site mutant SMAD4 genes. Bioinformatic analysis using GPS-SUMO (sumosp.biocuckoo.org) identified a conserved Ψ-K-x-E SUMOylation motif at SMAD4 Lys159. Combined with previously reported Lys113 modification,^39^ we generated arginine substitution mutants (K113R/K159R) through site-directed mutagenesis (Fig. 7A) and overexpressed them in human LECs (Fig. 7B and C). SUMO conjugation profiling revealed that the K113R mutation resulted in reduced SUMO1 binding but did not affect SUMO2/3 binding. K159R mutation abrogated both SUMO1 and SUMO2/3 conjugation (Fig. 7B). Nuclear trafficking analysis by immunofluorescence staining displayed remarkably reduced nuclear SMAD4 accumulation in both mutations, especially K159R (both *P* < 0.001 *vs*. WT) under TGFβ_2_ stimulation (Fig. 7D and E). In addition, double mutant (K113R + K159R) exhibited a significant reduction in EMT markers including fibronectin (*P* < 0.01), collagen I (*P* < 0.01), SLUG (*P* < 0.05), and SNAIL (*P* < 0.05) under TGFβ_2_ stimulation compared with WT SMAD4 in LECs (Fig. 7F and G).

## Discussion

To our knowledge, our study provides the first evidence that SUMOylation critically promotes LEC EMT and ASC pathogenesis. The study reveals that SUMOylation E1 enzymatic activity facilitates LEC proliferation and invasion, and EMT progression. SMAD4, a critical mediator of TGFβ signaling, undergoes SUMOylation during fibrotic transformation. SUMOylation governs TGFβ signal intensity by modulating SMAD4's nucleocytoplasmic shutting and intranuclear stability. Therapeutic intervention studies demonstrate that pharmacological inhibition of SUMO E1 (ML792) abrogates SMAD4 SUMOylation and suppresses TGFβ_2_-driven EMT in human LECs and attenuates fibrotic plaque formation in experimental ASC models. The findings establish SUMOylation E1 blockade as a novel therapeutic strategy to mitigate lens capsular fibrosis.

Previous investigations have established SUMOylation's regulatory role in vertebrate ocular development, particularly in retinal differentiation and lens morphogenesis.^23^ Notably, during lens development, SUMO1 and SUMO2/3 exhibit spatiotemporal specificity in murine LECs versus lens fiber cells at embryonic day 16.5. These paralogs also demonstrate opposing regulatory effects on the lens differentiation process.^23^ Intriguingly, our findings show that SUMO1 deficiency exerts no discernible impact on retinal/lens development or epithelial marker (E-cadherin), aligned with established developmental biology literature documenting effective compensation by SUMO2/3 paralogs in maintaining critical developmental processes.^40^^,^^41^ This functional redundancy underscores the evolutionary conservation of SUMOylation networks while highlighting subtype-specific regulatory complexity within the SUMO family.

Emerging experimental and clinical evidence furtherly underscores SUMOylation's pathophysiological relevance in ocular diseases, particularly ocular tumors,^42^^,^^43^ diabetic retinopathy,^44^^,^^45^ corneal endothelial injury,^46^ and senile and secondary cataractogenesis.^36^ Notwithstanding the established role of SUMOylation in lens development and senescence, its regulatory involvement in lens capsular fibrotic pathologies remains previously uncharted. Our study addresses this critical knowledge gap through a systematic investigation of SUMOylation alteration in fibrotic cataractogenesis. Evidence in our study revealed that human ASC specimens exhibited marked elevation of SUMO1- and SUMO2/3-mediated SUMOylation compared with non-fibrotic controls. SUMO1/2/3 overexpression induced mesenchymal transition in human LECs. However, genetic knockdown targeting individual SUMO paralog may inadvertently up-regulate compensatory SUMOylation mediated by other family members, resulting in incomplete suppression of both EMT and capsular fibrogenesis. The results imply the necessity for a comprehensive, systems-level approach to effectively regulate the SUMOylation process.

The SUMOylation cascade, analogous to ubiquitination, progresses through three enzymatically coordinated steps. Briefly, mature SUMO, via the C-terminal diglycine motif, undergoes ATP-dependent activation by the SAE1/UBA2 heterodimer at the initial stage. Then, the activated SUMO is transferred to the sole E2 enzyme UBC9, followed by substrate-specific modification by E3 ligases.^11^, ^12^, ^13^, ^14^ Notably, only one E1 complex (SAE1/UBA2) and one E2 enzyme (UBC9) mediate this pathway across species, contrasting with ubiquitination's multigenic enzyme repertoire. Clinical studies implicate SUMO E1 overexpression in chemotherapy resistance and poor prognosis across malignancies (glioma, hepatocellular carcinoma, and breast cancer),^31^^,^^47^^,^^48^ suggesting SUMO E1 suppression as a viable therapeutic strategy.

Our experimental findings extend this paradigm to ocular fibrosis. We found SAE1/UBA2 co-up-regulation in LECs of injury-induced murine ASC, indicating a positive association between SUMO E1 level and lens fibrosis. SAE1/UBA2 drove hyper-proliferation and enhanced invasion and mesenchymal transition of LECs. Efficient knockdown of SAE1/UBA2 could eliminate TGFβ_2_-induced LEC EMT. Complete SAE1/UBA2 depletion in LEC was lethal, while partial gene knockdown failed to block TGFβ_2_-induced EMT in our study (data not shown), probably due to residual E1 activity despite protein reduction. This phenomenon underscores the necessity for pharmacological E1 inhibition rather than partial genetic suppression to abrogate SUMOylation-dependent fibrosis. The enzymatic singularity of SUMO E1 complex (SAE1/UBA2) positions it as a high-value therapeutic target for lens fibrosis intervention.

Current pharmacological strategies focus on three classes of inhibitors as natural compounds: ginkgolic acid and anacardic acid (non-selective SUMOylation suppression)^49^; synthetic small molecules: ML792 (covalent adduct formation with activated SUMO) and TAK-981 (ML792 derivative, clinical stage)^50^^,^^51^; biosynthetic agents: Davidiin and kerriamycin B (E1 complex destabilizers).^52^^,^^53^ Natural resource-derived inhibitors can have several non-SUMO-related effects in cells. ML792 demonstrated unparalleled therapeutic potential due to its dual isoform inhibition for SUMO1/2/3 and covalent binding specificity to minimize off-target effects.^50^ ML792 and its pharmacologically optimized compound TAK-981 exhibit a remarkably inhibitory effect on tumor cell proliferation and metastasis, and have entered phase I clinical trials in metastatic malignancies^54^, ^55^, ^56^, ^57^, ^58^, ^59^, ^60^ (ClinicalTrials.gov, Identifier: NCT03648372, NCT04065555, NCT04074330, and NCT04381650). Besides, ML792 and TAK-981 have recently demonstrated preclinical efficacy in enhancing dexamethasone sensitivity.^57^^,^^61^^,^^62^ Synergistic combination therapy with glucocorticoids presents promising therapeutic potential to suppress inflammation and residual LEC EMT, aiming to abrogate lens capsular fibrosis.

Given this research context, we evaluated the therapeutic potential of both ginkgolic acid and ML792 in LEC EMT. Both compounds exhibited high pathway specificity, effectively suppressing global SUMOylation without altering acetylation or ubiquitination in human LECs. To furtherly confirm the SUMOylation-dependent mechanism underlying ML792-mediated EMT suppression, we overexpressed SENP1, one of the specific deSUMOylation enzymes, in human LECs. SENP1 overexpression significantly abrogated TGFβ_2_-induced EMT markers and SMAD4 nuclear trafficking in a manner phenocopying ML792 treatment. Our findings align with established literature demonstrating that SENP family members, particularly SENP1 and SENP2, regulate SMAD4 deSUMOylation, nuclear translocation, and EMT suppression in intestinal fibrosis models.^63^^,^^64^ This consensus further validates our hypothesis that SUMOylation constitutes a critical regulatory mechanism governing SMAD4 functionality and confirms that the anti-EMT efficacy of E1 inhibitors originates specifically from SUMO pathway inhibition rather than off-target effects. Notably, in our LEC system, single SENP1 knockdown exhibited limited efficacy in attenuating TGFβ_2_-induced EMT markers, suggesting functional redundancy among SENP isoforms. This compensatory phenomenon is further complicated by reports that specific SENP family members may exhibit pro-EMT activity, as observed in tumor progression models.^65^^,^^66^ Collectively, these observations highlight that the therapeutic window of SUMO pathway intervention requires careful evaluation of tissue-specific SENP functions, and pharmacological SUMOylation inhibitors provide more robust EMT modulation than isoform-specific SENP targeting.

Crucially, ML792 demonstrated superior efficacy both on SUMOylation suppression and LEC EMT modulation, relative to ginkgolic acid, regardless of TGFβ-stimulation status. Subsequently, ML792 was employed to inhibit global SUMO conjugation in TGFβ_2_-cultured rat lens and injury-induced mouse ASC model. Notably, ML792 demonstrated remarkable efficacy, achieving near-complete inhibition of TGFβ_2_-induced lens fibrosis in rat models and significantly attenuating ASC progression in murine models. Importantly, toxicological evaluation revealed no evidence of acute toxicity in LECs or systemic toxicity in treated mice. These findings provide compelling preclinical evidence supporting the therapeutic potential of SUMO E1 inhibitor for preventing lens fibrotic pathologies.

While our results clearly demonstrate functional involvement of SUMO modification in lens fibrosis, the specific molecular target(s) within this pathway remain to be identified. The SUMO modification system interacts with multiple signaling pathways, including TGFβ signaling, to regulate EMT in pathological contexts ranging from bladder cancer and hepatocellular carcinoma to cardiovascular fibrosis.^67^, ^68^, ^69^, ^70^ Given the well-documented role of TGFβ/SMAD signaling as the canonical driver of EMT in lens fibrosis,^2^^,^^10^^,^^38^^,^^71^^,^^72^ coupled with emerging evidence of SUMOylation's regulatory role in fibrosis-related diseases,^73^, ^74^, ^75^ our study establishes a novel connection between these two pathways in lens pathology research.

The assembly of R-Smad–Smad4 complex and their nucleocytoplasmic trafficking constitute the central regulatory mechanism of TGFβ/SMAD signaling.^76^^,^^77^ To identify potential SUMOylation targets within this pathway, we systematically analyzed key SMAD family members in TGFβ_2_-stimulated human LECs treated with or without ML792. Intriguingly, neither total levels nor TGFβ_2_-induced phosphorylation status of SMAD2/SMAD3 showed ML792-dependent alterations. This pattern suggests that SUMOylation acts downstream of SMAD2/3 heterodimer formation and phosphorylation in the signaling cascade.

We therefore focused on SMAD4, a key signaling molecule containing lysine-rich domains with a conserved ψ-KxD/E motif, a canonical SUMOylation consensus sequence. Although SMAD4 is not strictly required for basal TGFβ signaling, it critically amplifies signaling responses by stabilizing DNA complexes, a mechanism conserved across TGF-β/activin/Nodal and bone morphogenetic protein (BMP) signaling branches of the TGFβ superfamily.^76^, ^77^, ^78^ Literature-supported evidence reveals that SMAD4 undergoes functional SUMOylation modification in various pathological conditions. UBC9-mediated SUMOylation enhances SMAD4 nuclear retention in human osteoblasts and cervical cancer cells.^79^^,^^80^ Protein inhibitor of activated STAT 1 (PIAS1)-catalyzed SUMO1 conjugation promotes SMAD4-driven metastasis in non-small cell lung cancer.^81^ RanGAP1-regulated SUMO1 modification modulates SMAD4 nucleocytoplasmic trafficking and nuclear accumulation during cutaneous fibrogenesis.^82^

In the ocular lens, SMAD4 is predominantly localized to the cytoplasm of LECs under physiological conditions. Nuclear translocation of SMAD4 becomes evident during the postoperative phase (10 days post-removal) in human cataract specimens.^71^ This spatial redistribution occurs more rapidly in pathological conditions, as demonstrated in the trauma-induced ASC murine model, where SMAD3/4 nuclear accumulation in adherent LECs manifests within 12 h of capsule injury.^83^ We hypothesize that the dynamic equilibrium between SMAD4 SUMOylation and deSUMOylation acts as a molecular switch, which governs TGFβ signaling under pathological stress conditions, including mechanical injury and inflammatory stimuli. Comprehensive data in this study validated that SMAD4 underwent post-translational modification by all SUMO paralogs (SUMO1/2/3).

Pharmacological inhibition with ML792 substantially attenuated SMAD4 SUMOylation levels and subsequently blocked SMAD4 nuclear translocation and EMT-associated gene expression. Unlike its reported efficacy in oral squamous cell carcinoma (5–10 μM),^84^ ginkgolic acid showed minimal SMAD4 SUMOylation inhibition in our system (Fig. S15). This discrepancy suggests potential tissue-dependent SUMO regulation requiring further study. In addition, site-directed mutation of predicted SUMOylation sites in SMAD4 abrogated TGFβ_2_-induced EMT in LECs. This functional ablation establishes SUMO modification of SMAD4 as an essential regulatory mechanism governing fibrotic transformation in lens pathology. Our findings provide novel mechanistic insights into the regulation of lens capsular fibrosis and establish SMAD4 SUMOylation as a critical checkpoint in ocular fibrotic pathology.

Based on our study, ML792 demonstrated its advantage of more remarkable SUMOylation inhibition than gene approaches due to its consistent activity across experiments. Pharmacological targeting of SMAD4 SUMOylation through SUMO E1 inhibition demonstrates therapeutic promise for fibrotic lens disorders, including ASC, postoperative lens fibrosis, and postoperative capsular opacification. The clinical translation of ML792 still faces several pharmacological and biological challenges that require systematic resolution. These include pharmacodynamic validation of target engagement in ocular tissues; mitigation of potential off-target effects on non-SMAD4 SUMOylation substrates; optimization of ocular pharmacokinetics necessitating advanced delivery systems for sustained anterior segment bioavailability; development of patient stratification biomarkers for treatment-responsive populations; and long-term safety evaluation of SUMO pathway modulation. Addressing these requires combinatorial strategies integrating structure-based drug optimization, nanoparticle-mediated delivery platforms, and parallel development of SUMOylation-state biomarkers for precision dosing.

**Figure 8:**
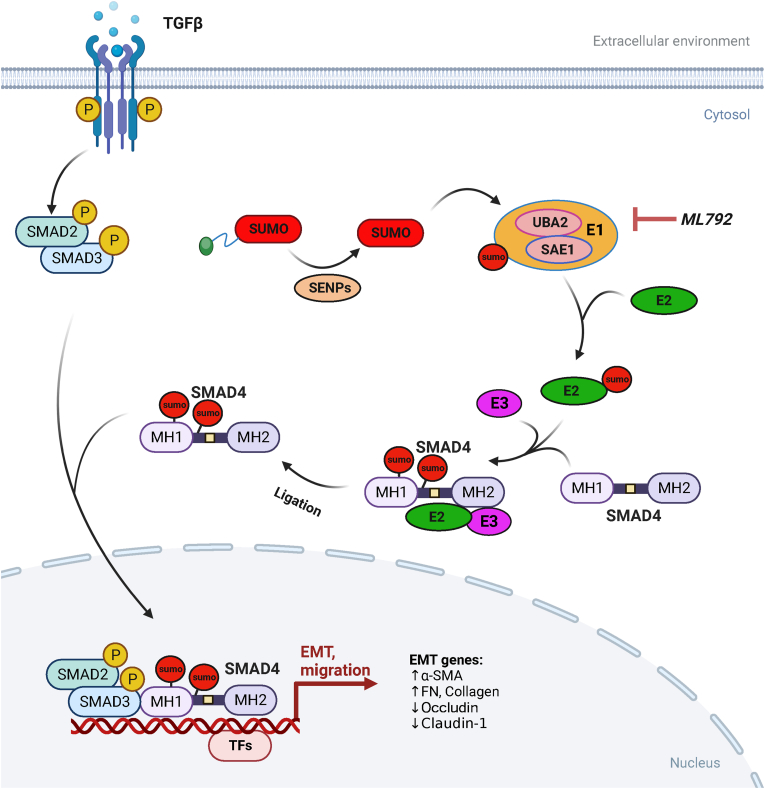
Mechanistic schema of SUMO E1-mediated SMAD4 SUMOylation in lens fibrogenesis. In TGFβ/SMAD signaling, the SAE1/UBA2 heterodimer (SUMO E1) catalyzes SMAD4 SUMOylation at Lys113/159 residues, enhancing nucleocytoplasmic trafficking efficiency of the SMAD complex. SUMOylated SMAD4 accumulates in the nucleus, stabilizing the transcriptional machinery of epithelial–mesenchymal transition (EMT)-related genes (*e.g.*, *SNAIL*, *SLUG*, *FN1*, *COL1A1*), thereby amplifying fibrotic gene expression. Sustained SUMOylation drives lens epithelial cell (LEC) transdifferentiation, characterized by α-SMA expression and extracellular matrix overproduction, culminating in anterior subcapsular cataract (ASC) progression. Pharmacological inhibition of SUMO E1 by ML792 blocks SMAD4 SUMOylation, disrupting nuclear translocation and abrogating pro-fibrotic transcriptional programs.

In conclusion, our findings establish SUMOylation as a critical pathogenic driver of LEC transdifferentiation and ASC. SUMO E1 enzyme activity facilitates LEC proliferation, migration, and EMT. Pharmacological inhibition of SUMO E1 via ML792 disrupts SMAD4 SUMOylation, thereby blocking nuclear translocation of SMAD complexes and subsequent fibrotic transformation (Fig. 8). These results delineate a SUMOylation-dependent regulatory axis in ocular fibrosis, where SMAD4 post-translational modification amplifies TGFβ/SMAD signaling through enhanced nucleocytoplasmic coordination. Our work not only elucidates a novel molecular paradigm in lens pathology but also reveals the therapeutic potential of SUMO pathway inhibition. The clinical translation of SUMO-targeting agents may pioneer a new therapeutic paradigm for preventing and treating fibrotic lens disorders.

## CRediT authorship contribution statement

**Min Hou:** Writing – original draft, Software, Methodology, Investigation, Data curation. **Yujie Ding:** Methodology, Investigation, Data curation. **Xuan Bao:** Data curation. **Liangping Liu:** Validation, Data curation. **Yulan Wang:** Writing – review & editing, Validation. **Mingxing Wu:** Writing – review & editing, Validation, Supervision, Funding acquisition, Formal analysis, Conceptualization.

## Data availability

All the original data generated or analyzed during this study are available upon reasonable request.

## Funding

This study was supported by the 10.13039/501100001809National Natural Science Foundation of China (No. 81970783), Science and Technology Program of Guangzhou, China (No. SL2024A03J00523), and the Youth Program of the National Natural Science Foundation of China (No. 82305319).

## Conflict of interests

The authors declared no conflict of interests.
