# Supplementary material for: Blockage of SUMO E1 enzyme inhibits ocular lens fibrosis by mediating SMAD4 SUMOylation
Source: Genes Dis. 2025 Aug 28;13(3):101827. doi: 10.1016/j.gendis.2025.101827 (PMC12886541; doi:10.1016/j.gendis.2025.101827)
Supplement: Multimedia component 1 [file mmc1.docx]

**Supplementary Data**

**1. Supplementary Tables**

**Table S1. Oligos Used for Gene Overexpression/Mutation**

| **Oligos** | **Sequences** |
| --- | --- |
| SUMO1-F | GGggtaccGATGTCTGACCAGGAGGCAAA |
| SUMO1-R | GCtctagaCAATTCCGTTTTGAACACCA |
| SUMO2-F | GGggtaccGATGGCCGACGAAAAGCCCAA |
| SUMO2-R | GCtctagaAGTCAGGATGTGGTGGAACC |
| SUMO3-F | GGggtaccGATGTCCGAGGAGAAGCCCAA |
| SUMO3-R | GCtctagaACGGGCCCTCTAGAAACTGT |
| SAE1-F | CCGgaattcATGGTGGAGAAGGAGGAGGC |
| SAE1-R | CCGctcgagCTTGGGGCCAAGGCACTCCA |
| UBA2-F | CCGgaattcATGGCACTGTCGCGGGGGCT |
| UBA2-R | CCGctcgagATCTAATGCTATGACATCAT |
| SENP1-F | CCCaagcttATGGATGATATTGCTGATAG |
| SENP1-R | GCtctagaTCACAAGAGTTTTCGGTGGA |
| SMAD4-F | CCGgaattcATGGACAATATGTCTATTAC |
| SMAD4-R | CCGctcgagGTCTAAAGGTTGTGGGTCTG |
| SMAD4-K113R-F | TTTTAAGTCAAACGCATACTGACAATATCTAACATGTTTTAGTTCATTTTTGTGAAG |
| SMAD4-K113R-R | CTTCACAAAAATGAACTAAAACATGTTAGATATTGTCAGTATGCGTTTGACTTAAAA |
| SMAD4-K159R-F | GCACATATTCATCCCTCACCATCATACTTGATGGAGCA |
| SMAD4-K159R-R | TGCTCCATCAAGTATGATGGTGAGGGATGAATATGTGC |

All target genes were annotated according to the Homo sapiens reference genome assembly.

**Table S2. Oligos Used for Gene Knockdown**

| **Oligos** | **Sequences** |
| --- | --- |
| 1-SAE1-shRNA-F | CCGGgctatgttggtcctttgtttaCTCGAGtaaacaaaggaccaacatagcTTTTTG |
| 1-SAE1-shRNA-R | AATTCAAAAAgctatgttggtcctttgtttaCTCGAGAtaaacaaaggaccaacatagc |
| 2-SAE1-shRNA-F | CCGGgcatgagtttgtagaggagaaCTCGAGttctcctctacaaactcatgcTTTTTG |
| 2-SAE1-shRNA-R | AATTCAAAAAgcatgagtttgtagaggagaaCTCGAGAttctcctctacaaactcatgc |
| 1-UBA2-shRNA-F | CCGGgcaccagatgtccaaattgaaCTCGAGttcaatttggacatctggtgcTTTTTG |
| 1-UBA2-shRNA-R | AATTCAAAAAgcaccagatgtccaaattgaaCTCGAGAttcaatttggacatctggtgc |
| 2-UBA2-shRNA-F | CCGGgctgtattgaaagtaggaataCTCGAGtattcctactttcaatacagcTTTTTG |
| 2-UBA2-shRNA-R | AATTCAAAAAgctgtattgaaagtaggaataCTCGAGAtattcctactttcaatacagc |
| Mock-shRNA-F | CCGGaagctggagtacaactacaacCTCGAGgttgtagttgtactccagcttTTTTTG |
| Mock-shRNA-R | AATTCAAAAAaagctggagtacaactacaacCTCGAGgttgtagttgtactccagctt |
| 1-siSUMO1 | GTTCCAATGAATTCACTCA |
| 2-siSUMO1 | CACTCAGGTTTCTCTTTGA |
| 3-siSUMO1 | GTGTTCCAATGAATTCACT |
| 1-siSUMO2 | GCATACACCACTTAGTAAA |
| 2-siSUMO2 | GGAGGATGAAGATACAATT |
| 3-siSUMO2 | AGCAGACGGGAGGTGTCTA |
| 1-siSUMO3 | ACACCATCGACGTGTTCCA |
| 2-siSUMO3 | GTGGTGCAGTTCAAGATCA |
| 3-siSUMO3 | AGCCAATCAATGAAACTGA |
| 1-siSENP1 | GGATGAAGTTCTCAGTGAA |
| 2-siSENP1 | GAAGATGAATTTCCTGAAA |
| 3-siSENP1 | GGACCAGCTTTCGCTTTCT |

All target genes were annotated according to the Homo sapiens reference genome assembly.

**Table S3. Oligo Primers Used for real-time quantitative RT-qPCR**

| **Gene** | **Forward primer** | | **Reverse primers** |
| --- | --- | --- | --- |
| SAE1 | TGGGTATTAGTCCTGACCTGCTTCC | CTCCAACCACCGCACACACTG | |
| UBA2 | CTCAGCCCTCCACCTCCACAG | ATTTCCTCTTGCGGCTTCTCTCTTC | |
| SUMO1 | TTGGACAGGATAGCAGTGAGATTC | ATCTTCTTCCTCCATTCCCAGTTC | |
| SUMO2 | CCGACGAAAAGCCCAAGGAAGG | AACTGCACCACAGAACCATCCTG | |
| SUMO3 | CGAGAGGCAGGGCTTGTCAATG | GGTGTCCTCGTCCTCCATCTCC | |
| SENP1 | TCGCCTGACCATTACACGCAAAG | GCACACTTGGCAAGCCCTTCTC | |
| β-Actin | TGGCACCCAGCACAATGAA | CTAAGTCATAGTCCGCCTAGAAGCA | |

All target genes were annotated according to the Homo sapiens reference genome assembly.

**Table S4. Antibodies Used in the Study**

| **Antibody** | **Application** | **Cat No.** |  | **Antibody** | **Application** | **Cat No.** |
| --- | --- | --- | --- | --- | --- | --- |
| anti-Fibronectin | WB, IHC | Abcam, #2413 |  | anti-SMAD4 | WB, IF | CST, # 46535 |
| anti-Col-IV | WB | Abcam, # 6586 |  | anti- SAE1 | WB | ABclonal, #  A0891 |
| anti-Col1A | WB | CST, # 91144 |  | anti- UBA2 | WB | CST, #8688 |
| anti-α-SMA | WB, IF | CST, # 48938 |  | anti-GFP | WB | Proteintech #66002-1-Ig |
| anti-α-SMA | IHC | Abcam, # 7817 |  | anti-HA | WB, IP | CST, #3724 |
| anti-ZO-2 | Wes | CST, # 2847 |  | anti-Flag | WB, IP | Sigma, #F1804 |
| anti-E-cadherin | WB, Wes | CST, # 3195 |  | anti-Flag | WB | CST, #14793 |
| anti-Claudin-1 | WB | CST, # 13255 |  | anti-SUMO1 | WB, IP, IF | Sigma #S8070 |
| anti-Occludin | WB | CST, # 91131 |  | anti-SUMO1 | IP, IHC | Santa, Cruz #SC-5308 |
| anti-SNAIL | WB, Wes | CST, # 3879 |  | anti-SUMO2/3 | WB, IF | Abcam, #81371 |
| anti-SLUG | WB | CST, # 9585 |  | anti-SUMO2/3 | IB | Abcam, #3742 |
| anti-CDK2 | WB | CST, # 2546 |  | anti-SENP1 | WB | CST, #11929 |
| anti-CDK4 | WB | CST, # 2906 |  | anti-β-Actin | WB | CST, #3700 |
| anti- p21Waf1/Cip | WB | CST, # 2947 |  | anti-β-Tubulin | WB | Proteintech, #10094-1-AP |
| anti- p27 Kip1 | WB | CST, # 3686 |  | anti-α-Actinin | WB | Proteintech, #11313-2-AP |
| anti- PCNA | WB | Proteintech # 10205-2-AP |  | Anti-GAPDH | WB | Proteintech, #60004-1-Ig |
| anti-Cyclin D1 | WB | Proteintech # 11554-1-AP |  | Normal rabbit IgG | IP | CST, #2729 |
| anti-Cyclin E1 | WB | Proteintech # 11554-1-AP |  | mouse IgG | IP | CST, #53484 |
| anti-Lamin A/C | WB, IF | CST, # 46535 |  | Anti-rabblit IgG | IF | CST, #8889 |
| anti-p-SMAD2 | WB | CST, #18338 |  | Anti-rabblit IgG | IF | CST, #4412 |
| anti- SMAD2 | WB | CST, #5339 |  | Anti-rabblit IgG | IF | Abcam, #150113 |
| anti-p-SMAD3 | WB | CST, #9520 |  | HRP-linked anti-mouse IgG | WB | CST, #7076 |
| anti-SMAD3 | WB | CST, #9523 |  | HRP-linked anti-rabbit IgG | WB | CST, #7074 |

**2. Supplementary Figures and Legends**

**Fig. S1 Global SUMOylation is enhanced in SUMO isoform-overexpressed LECs**

A-C. Immunoblot analysis of SUMOylation patterns in FHL124 LECs 24h post-transfection with empty vector (pCDNA3.1-3xHA), HA-tagged SUMO1, SUMO2 and SUMO3. Blots probed with: anti-HA antibody (exogenous SUMO conjugates), SUMO1 and SUMO2/3 antibodies.

D-E. Quantification analysis of global exogenous SUMO-conjugates in panel A and total SUMO-conjugates in panel B and C. One-way ANOVA with Bonferroni correction. ns, not significant, * p < 0.05, *** p < 0.001.

**Fig. S2 *Sumo1* deficiency does not affect eye and lens development in mice.**

A. Genotype validation. PCR and agarose gel electrophoresis analysis of tail DNA from S129 mice using primers: Forward-5’-CACCTGCCTCTACCTCAA-3’, Reverse-5’-TTCCCACAACTATTACCACT-3’.

B. Immunohistochemistry staining of SUMO1 protein in eyeball section of wild type (WT) and *Sumo1*^-/-^ mouse. Scale bar: 200μm.

C. Hematoxylin & eosin staining shows lens and retinal structure of WT and *Sumo1*^-/-^ mice. Scale bar: 200 μm.

D**.** WES analysis of E-cadherin protein in lens epithelium of WT and *Sumo1*^-/-^ mice. Bottom: quantification of E-cadherin normalized to GAPDH. Unpaired Student’s t test, ns, not significant, n=6 capsules/group.

**Fig. S3 SUMO isoform-specific deficiency may cause compensatory increase of other SUMO conjugates**

1. SiRNA-mediated SUMO isoform knockdown in FHL124 LECs. Cells transfected with non-targeting siRNA (Mock) or three SUMO1/2/3-specific siRNAs for 24h. Real-time qPCR analysis of SUMO isoform genes. One-way ANOVA with Bonferroni post-hoc test. ****P* < 0.001.

B-E. Immnublot analysis of global SUMOylation in engineered cells. Cells transfected with non-targeting siRNA (Mock) or validated SUMO1/2/3-specific siRNAs for 24h, followed by TGFβ_2_ (10 ng/mL) treatment for 24h. B, D. Immunoblots show SUMO1- and SUMO2/3- conjuagtes. C, E. Quantification of panel B and D normalized to β-Tubulin. One-way ANOVA followed by Bonferroni post-hoc test. ns, not significant, * p < 0.05, ** p < 0.01.

**Fig. S4 Verification of SAE1/UBA2 gene overexpression/knockdown in human LECs.**

A-B. FHL124 LECs was established to overexpress empty vector control, Flag-tagged SAE1 and Flag-tagged UBA2 via lentivirus infection plasmid system. Real-time qPCR analysis of SAE1/UBA2 gene expressions. Unpaired Student’s t-test, *** p < 0.001.

C-F. ShRNA-mediated SAE1/UBA2 knockdown in FHL124 LECs with two strands of shRNA separately. C, D. Real-time qPCR analysis of SAE1/UBA2 gene expressions. One-way ANOVA with Bonferroni post-hoc test. ***p < 0.001. E, F. Immunoblots show SAE1/UBA2 protein expressions followed by densitomentric quantification analysis normalized to α-Actinin and β-Tubulin. One-way ANOVA with Bonferroni correction. ** p < 0.01, *** p < 0.001.

**Fig. S5 SAE1/UBA2 knockdown suppresses global SUMOylation**

FHL124 LECs transduced with scrambled shRNA (Mock) or shRNAs targeting SAE1/UBA2 subunits of the SUMO E1 enzyme complex were cultured and stimulated with 10 ng/mL TGFβ_2_ for 24 hours. A, C. Immunoblot show global SUMO1 conjugates and SUMO2/3 conjugates levels. B, D. Quantification analysis of panel A and C normalized to β-Tubulin. One-way ANOVA with Bonferroni correction. * p < 0.05, ** p < 0.01, *** p < 0.001.

**Fig. S6** **Overexpression of SAE1/UBA2 does not alter vertical migration of human LECs**.

1. FHL124 LECs stably expressing empty vector, flag-tagged SAE1 and flag-tagged UBA2 were subjected trans-well migration assays. 2.5x10^4^ cells in serum-free DMEM medium were loaded to trans-well chamber inserts. After 24 h incubation, migrated cells were imaged using phase-contrast microscopy. Representative images shown with scale bar, 200 μm.

B. Quantification and statistical analysis of migration cell in panel A. One-way ANOVA with Bonferroni post-hoc test. ns, not significant (p = 0.529).

**Fig. S7 Pharmacological inhibition of SUMO E1 does not alter global acetylation/ubiquitination in TGFβ_2_-induced LECs**

A-D. FHL124 LECs were treated with: 0.1%DMSO, 10μM ML792, 10μM ginkgolic acid (GA) ± 10 ng/mL TGFβ_2_ for 24h. Immunoblot analysis of global acetylated proteins and ubiquitin conjugates in the six groups of LECs. Quantification normalized to GAPDH and β-Tubulin. One-way ANOVA analysis. ns, not significant.

E. FHL124 LECs were grouped and treated as blank, vehicle (0.1%), ML792 (10μM). CCK-8 assay was performed at for 0h, 24h and 48, respectively. OD 450 values were statistical analyzed to assess cell proliferation. One-way ANOVA with Bonferroni post-hoc comparison. ns, not significant, * p < 0.05.

**Fig. S8 SENP1-mediated deSUMOyltion reduces EMT markers and TGFβ_2_-induced SMAD4 nuclear translocation in LECs**

1. G. Human FHL124 LECs was transfected with Flag empty vector or Flag-tagged SENP1 for 24h. A. Immunoblot analysis of flag, SENP1, epithelial and EMT marker expression levels. B, C. Quantification of panel A normalized to β-Actin. Unpaired Student’s t-test. ** p < 0.01, *** p < 0.001. D, E. Immunoblots show global SUMO1- and SUMO2/3-conjugates. F. Quantification analysis of panel D and E normalized to β-Actin and β-Tubulin. Unpaired Student’s t-test. * p <0.05. *** p < 0.001.

G. Immunofluorescence staining of SMAD4 (green) and DAPI (nuclei, blue) in LECs transfected with Flag empty vector or Flag-tagged SENP1, followed by 10 ng/mL TGFβ_2_ treatment for 2h.

H. Quantification of nuclear SMAD4 fluorescence intensity from panel G. One-way ANOVA with Bonferroni correction. * p < 0.05, *** p < 0.001.

**Fig. S9 SENP1 gene knockdown fails to inhibit TGFβ_2_ induced LEC EMT.**

A. SiRNA-mediated SENP1 knockdown in FHL124 LECs. Cells transfected with non-targeting siRNA (Mock) or three strands of SENP1-specific siRNAs for 24h. Real-time qPCR analysis of SENP1 gene expression. One-way ANOVA with Bonferroni post-hoc test. *** p < 0.001.

B. Cells transfected with Mock siRNA or validated one SENP1-specific siRNA for 24h, followed by TGFβ_2 (_10 ng/mL) treatment for 24h. Immunoblots show SENP1 and fibrotic markers.

C. Quantification of panel B normalized to β-Tubulin. Unpaired Student's t-test. ns, not significant, *** p < 0.001.

**Fig. S10** **ML792 eliminates global SUMOylation in cultured rat lens epithelium**

A-B. *Ex vivo* rat lens organ culture model: macroscopic lens opacity assessment after 7-day treatments: vehicle (0.1% DMSO), TGFβ_2_ (10ng/mL), ML792 (10 μM) and TGFβ_2_ +ML792. Immunoblot analysis of global SUMO1- and SUMO2/3-conjugates in lens epithelium. GAPDH and β-Tubulin loading control shown.

C. Densitometirc quantification of penal A and B. One-way ANOVA with Bonferroni correction. ** *P* < 0.01, ****P* < 0.001.

**Fig. S11 Systemic administration of ML792 does not cause obvious organ destruction in mice.**

Intraperitoneal(i.p.) injection of vehicleor ML792 (7.5mg/kg) was performed on C57/6J mice.

A. Representative pre-harvest images of indicative organs from C57BL/6J mice 7 days after i.p. injection of vehicle or ML792.

B. Quantification of tissue areas analyzed using ImageJ (n = 6 mice/group). Unpaired Student's t-test, ns. not significant.

C. Hematoxylin & eosin staining of eyeballs. Scale bar: 200 μm.

D. Hematoxylin & eosin staining of panel A indicated organs. Scale bar: 50 μm.

**Fig. S12 Pharmacological inhibition of SUMO E1 does not affect TGFβ_2_-driven** **SMAD2/3 phosphorylation.**

1. Immunoblot analysis of total SMAD2/3/4 and phosphorylated SMAD2/3 (p-SMAD2/3) in FHL124 LECs were cultured with vehicle (0.1% DMSO), TGFβ_2_ (10 ng/mL), ML792 (10 μM) and TGFβ_2_ +ML792 for 30min.

B. Quantification of panel A. One-way ANOVA followed by Bonferroni post-hoc comparison. ns, not significant, ***p < 0.001.

**Fig. S13 SMAD4 undergoes SUMO1/2/3-mediated SUMOylation in human LECs *in vitro.***

FHL124 LECs stably expressing flag empty vector or flag-tagged SMAD4 were transfected with HA-tagged SUMO1/2/3 for 24h.

A-C. Top：INPUT: Whole cell lysis was immunoblotted for SUMO1/2/3, Flag, and β-tubulin (loading control).

Bottom: IP: Anti-SUMO1 or HA antibody followed by anti-Flag immunoblotting (IB).

Reciprocal IP: Anti-Flag IP followed by anti-SUMO1/2/3 immunoblotting (IB).

**Fig. S14** **TGFβ_2_-driven SMAD4 nuclear dynamics in human LECs.**

Time-course immunofluorescence analysis of nuclear SMAD4 localization in LECs treated with 10 ng/mL TGFβ2 over 24 h. SMAD4 (red), DAPI (nuclei, blue) and F-actin (Phalloidin, green) at the time points. Scale bar: 20μm

**Fig. S15** **Pharmocological inhibitor ginkgolic acid (GA) fails to disrupt SMAD4 SUMOylation in TGFβ2-induced LECs.**

A-C. Immunoprecipitation (IP) of flag-tagged SMAD4 overexpressed LECs co-treated with 10 ng/mL TGFβ_2_ and 0.1% DMSO or 10 μM GA for 2 h: INPUT lysates: immunoblotted for SMAD4 and Flag (β-tubulin loading control). Flag IP: immunoblotted for SUMO1 and SUMO2/3.
